# P-892. Reality of Resistance Data: Practical Applications of Risk Adjusted Antimicrobial Resistance Data Using The National Healthcare Safety Network Antimicrobial Use and Resistance Module

**DOI:** 10.1093/ofid/ofaf695.1100

**Published:** 2026-01-11

**Authors:** Elizabeth Dodds Ashley, Jeannette Bouchard, Melissa D Johnson, April Dyer, Angelina Davis, Deverick J Anderson, Rebekah W Moehring

**Affiliations:** Duke Center for Antimicrobial Stewardship and Infection Prevention, Durham, NC; Duke Antimicrobial Stewardship Outreach Network, Elgin, SC; Duke University, Durham, North Carolina; Duke Center for Antimicrobial Stewardship and Infection Prevention, Durham, NC; Duke Center for Antimicrobial Stewardship and Infection Prevention, Durham, NC; Duke Center for Antimicrobial Stewardship and Infection Prevention, Durham, NC; Duke University, Durham, North Carolina

## Abstract

**Background:**

Beginning in 2024, US hospitals were required to participate in the CDC National Healthcare Safety Network (NHSN) antimicrobial use and resistance (AUR) module, which captures data on antimicrobial use (AU) and targeted antimicrobial resistance (AR) results. The AUR module outputs provide facility-level risk adjustment that enables inter-hospital benchmarking. While many stewards have experience using AU, end users are less familiar with AR module outputs. Also, national aggregate data reports of AR are not yet available. We describe an initial year of risk-adjusted output from the NHSN AR option for our network of 46 hospitals included in the Duke Antimicrobial Stewardship Outreach Network (DASON) NHSN Group.

**Methods:**

AR data for each facility were reported in monthly increments following NHSN protocol specifications. A dataset was generated and reports for two risk-adjusted AR metrics were extracted: standardized resistant infection ratio (SRIR) and pathogen-specific standardized infection ratio (pSIR) for 2024. These observed to expected (O:E) ratios are calculated for hospital onset (collected after day 3 of admission) isolates. Data were summarized using descriptive measures.

**Results:**

SRIRs were available for seven drug-resistant pathogen groups and three of the four anatomic sites captured in NHSN (blood, urine and lower respiratory tract). pSIRs were calculated for the same three sites for four pathogen groups. No risk adjusted metrics were available for cerebrospinal fluid specimens. Distributions for each of the 21 SRIR and 12 pSIR categories across 46 DASON hospitals (Tables 1 & 2) demonstrate great variability among hospitals.

Specifically, effects of small populations on predicted isolate numbers were common in our community hospital network. Of 966 predicted isolate calculations (SRIR denominators) for 2024, (531) 55% were predicted to be less than one. Similarly, for the 552 predicted hospital onset pathogens, 132 (24%) were predicted to be less than one.

**Conclusion:**

Early experience with AR data suggests that low predicted numbers of isolates may result in initially high observed to expected ratios. More experience on how to use and interpret hospitals’ AR risk-adjusted metrics in routine stewardship and prevention work is needed.

**Disclosures:**

Elizabeth Dodds Ashley, PharmD, MHS, HealthtrackRx: Advisor/Consultant|UpToDate, Inc.: Author Royalties Melissa D. Johnson, PharmD MHS AAHIVP, Biomeme: Licensed technology, method to detect fungal infection|Biomeme: Licensed technology, method to detect fungal infection|Scynexis: Grant/Research Support|Scynexis: Grant/Research Support|UpToDate: Author Royalties|UpToDate: Author Royalties Rebekah W. Moehring, MD, MPH, FIDSA, FSHEA, UpToDate, Inc.: Author Royalties

